# Action-Centered Exposure Therapy (ACET): A New Approach to the Use of Virtual Reality to the Care of People with Post-Traumatic Stress Disorder

**DOI:** 10.3390/bs8080076

**Published:** 2018-08-16

**Authors:** Sorelle Audrey Kamkuimo Kengne, Mathilde Fossaert, Benoît Girard, Bob-Antoine J. Menelas

**Affiliations:** 1 Department of Computer Sciences and Mathematics, University of Quebec at Chicoutimi, 555 Blv Universite, Chicoutimi, QC G7H 2B1, Canada; mathilde.fossaert@gmail.com; 2 La Futaie: Therapy Center, 1061, Boulevard Tadoussac, Saint-Fulgence, QC G0V 1S0, Canada; beni.girard@gmail.com

**Keywords:** ACET, PTSD, exposure therapy, virtual reality, learning, action, interactivity

## Abstract

Post-Traumatic Stress Disorder (PTSD) can be seen as the result of dysfunctional beliefs that associate stimuli with a danger or a threat leading to anxious reactions. Exposure therapy is so far considered to be the most effective treatment, and research suggests that it is mainly based on a habituation process. Based on learning theories, it appears that a passive systemic exposure to traumatic stimuli should not be the best option for the treatment of PTSD. We hypothesis that an active learning of safer and healthier coping strategies combined with systematic exposure should be more effective in reducing the psychological distress associated with PTSD. In this paper, we describe the theoretical foundations of this approach that focuses on the action and activity of the patient in his or her exposure environment. In this approach, we take advantage of Virtual Reality technologies and learning mechanics of serious games to allow the patient to learn new safe associations while promoting the empowerment. We named this action-centered exposure therapy (ACET). This approach exploits behaviorism, cognitivism, and constructivism learning theories. With the different benefits of virtual reality technologies, this approach would easily integrate with in-virtuo exposure therapy and would allow us to exploit as much as possible the enormous potential of these technologies. As a first step toward validation, we present a case study that supports the ACET approach.

## 1. Introduction

According to the DSM-V diagnostic criteria [1], PTSD is the result of a trauma: an experience that has threatened the health or well-being of a person [2]. The reliving of this situation through intrusive memories, nightmares, or flashbacks is a manifestation of PTSD, and the individual adopts avoidance behaviors regarding trauma-related stimuli. Moreover, the individual may have negative alterations of cognition and mood as well as arousal symptoms such as aggression, sleeps disturbance, or hypervigilance [1]. 

Currently, there are two main approaches to the treatment of PTSD: psychotherapy and pharmacotherapy [3] (p. 1346). Psychotherapy takes several forms, including anxiety management, cognitive therapy, psychoeducation, play therapy, and exposure therapy [4]. Pharmacotherapy consists primarily of administering antidepressants to the patient. Although it may have side effects [3] (p. 1349), it is generally used to enhance psychotherapy efficacy [5]. A systematic review and meta-analysis revealed the superiority of trauma-focused psychotherapy (TFP) over medication [6] and suggests using the exploitation of TFP as a first-line PTSD treatment. Prolonged exposure therapy [7] is strongly recommended by the American Psychological Association [8] based on concrete scientific evidence [9,10].

Exposure therapy relies on the Pavlovian conditioning principle that “fears could be both conditioned and extinguished through learning experiences” [11]. Exposure therapy can be done in several ways: imaginal exposure, in vivo exposure and in-virtuo exposure. In-vivo exposure therapy involves confronting the patient with his anxiety in the real world [12]. However, there are limitations in the equipment used and in the cost of the therapy [13] (p. 68). Moreover, it is often impossible to reproduce certain events like a car accident or war situations. In imaginal exposure therapy, the therapist guides the patient to remember the anxiety-provoking situation in order to gradually reduce its impact [14]. Media supports, such as photos or videos, are often exploited to enhance the patient’s imagination. In this approach, the main limitation remains the difficulty and the willingness of the patient to remember the traumatic situation [15,16]. 

For in-virtuo exposure therapy, virtual reality (VR) technologies are exploited to “create a safe virtual environment (VE) with the ability to allow patients to “relive” their experiences in a thorough and efficient method by slowly exposing them to the exact or similar events that cause anxiety” [13] (p. 214). With the same or greater efficacy as other exposure therapies [17], in-virtuo exposure saves time and money [13] (pp. 265–283). It allows for reproducing complex and dangerous events like combat situations [18]. This therapy is interactive, controlled, secured, confidential, and customizable for each patient [19,20,21]. The intensity of the exposure can be adapted to the anxiety level of the patient [22,23], and it is possible to repeat a specific task as many times as necessary without loss of time and means [24,25,26]. Given these advantages, in-virtuo exposure therapy could be a good alternative for patients who are resistant to traditional treatment [17]. 

In-virtuo therapy, as well as exposure therapy for the treatment of PTSD has evolved supported by concrete evidence [9,27]. Researchers suggest that the main mechanism behind the effectiveness of exposure is an inhibitory learning model of extinction [28] and propose optimization strategies [29,30]. However, from [31], we noticed that the treatment does not necessarily involve a systematic unlearning of irrational associations but rather a new learning of safe associations from the patient. As seen in the case of arachnophobia, a more effective and successful therapy could occur through an emphasis on the generalization of skills learned during therapy to life after therapy [13] (p. 63). As emphasized in [32]: “To be successful, meaningful and lasting, learning must include all three of these crucial factors: activity (practice), concept (knowledge) and culture (context)” [33]. We assume that an active learning of safer and healthier coping strategies combined with systematic exposure should be more effective in reducing the psychological distress associated with PTSD [22]. This vision of active learning could easily fit into an in-virtuo context because VR technologies offer a means to address most sensory-motor capabilities [34] achieved through interactivity [35]. This may also be reinforced using recent advances in Serious Games (SGs). Today, SGs are mainly computer-based games whose chief task is not entertainment. Nevertheless, having the game context let to produce an environment that has the potential to motivate the player and to engage him with the content [36]. 

Hence, using VR technologies and SG mechanics, we want to propose a new approach—action-centered exposure therapy (ACET)—for the treatment of people suffering from PTSD. Although for many years VR has been used in this area, it is important to note that this use has focused on the passive systematic exposure of patients to their traumatic experiences. The goal is to allow them, by habituation, to learn to control their emotional reactions [35]. However, Foa et al. have gone beyond habituation with the use of prolonged exposure therapy [7]. In the same way, we want to exploit advanced interactions and gamification techniques [37] to allow the user to be active in a learning-centered environment. Therefore, in the ACET approach, the exploitation of VR is different. Here the focus is the action and interactivity of the patient in the VE. The aim is to allow the patient to relive the traumatic situation differently in order to build a new reality. Although ACET uses VR to expose the patient to stimuli that may cause anxiety, since this exposure is centered on his action, this approach will, by means of learning, lead the patient to build new and healthy associations with the anxiogenic stimuli.

The paper is structured as follows. In the next section, we analyze the learning process to highlight the importance of action in learning. Section 3 presents our proposed approach, and to reinforce the validity of our approach, in Section 4, we summarize an empirical case supporting it.

## 2. Relationship between Learning and PTSD 

In this section, we first examine three main learning theories: behaviorism, cognitivist, and constructivist theories. Second, we discuss how the learning process may aid in the understanding of PTSD.

### 2.1. Learning Paradigms 

According to behavioral learning theory, a reaction becomes a reflex when the cerebral hemispheres create an association between a stimulus and said reaction [38]. In this way, the acquisition of new habits and behaviors follows a succession of stimuli-response processes [39]. Seen from an operant conditioning perspective, the stimulus can also be based on an action of the subject that may or may not trigger the desired response. In this sense, learning is an exploration and discovery process in which the subject tends to repeat actions that produce positive responses while avoiding others [40] (pp. 51, 52, 57).

In contrast, the cognitivist and constructivist learning paradigms are not limited to stimuli and responses but also consider learning as a task of information-processing based on cognitive functions (perception, attention, memory, language, and intellectual activities) [41]. During the learning process, this information processing considers the state of knowledge of the subject. The subject uses the organized mental image that he has already established to represent the current situation [42,43]. At the end of the learning process, he has a new representation that he can reuse in future situations. Thus, the subject constructs his own knowledge. Although the presence of a supervisor is necessary [44], the learner is autonomous. He learns by doing activities that require reflection, reasoning, action, and decision making [45] by exploring, manipulating, and modifying his environment through his actions [46,47]. Therefore, learning is a process of acquiring experience [48] and also a process of creation [23] that involves engagement and involvement in specific tasks linked to the learning purpose [49]. 

### 2.2. Dysfunctional Associations as Causes of PTSD

PTSD is the result of learning as dysfunctional beliefs that associate stimuli (e.g., event, place, situation, activity) with a danger, threat, or problem, which leads to anxious reactions [50] (pp. 4–6, 23, 493). As pointed out in [51,52], the experience of a traumatic situation creates a fear structure in the subject’s memory. This structure is represented as a fear network consisting of anxiety-provoking stimuli, responses to these stimuli, and meanings that the subject gives to these stimuli and these responses [53]. As a result, subjects tend to avoid anxiety-provoking elements for fear of activating the fear structure and eliciting negative responses (anxiety). In addition, mental activity and cognition are involved in the form of intrusive memories, nightmares, and flashbacks [1]. The individual victim of a trauma also tends to avoid the memories of that experience, but unfortunately, this mental activity amplifies symptoms by increasing related dysfunctional cognitions ([50] (p. 516), [53]).

### 2.3. Learning as the Main Mechanism of PTSD Treatment 

As mentioned in [31,54], the purpose of therapy is to create new safe associations between a formerly feared situation and the absence of fear. According to the emotional processing principle, the goal would be to access the patient’s fear structure by presenting anxiety-provoking stimuli and to then incorporate the corrective information [53]. Mechanisms involved in the therapy must allow for a dissociation of the stimuli from the fear responses and a modification of erroneous meanings that the patient gives to the elements contained in his fear structure [53]. Nevertheless, the treatment does not necessarily involve a systematic unlearning of irrational associations but rather a new learning of safe associations [31,53]. Following the logic of operant conditioning, which states that people tend to repeat rewarding actions, we assume that the patient can be expected to repeat actions that reduce anxiety. By combining this aspect with cognitivist and constructivist theories, there will be a positive modification of the patient’s behavior patterns across sessions as he acquires experience through his self-directed activities. At the end of the therapy, he can use his new non-problematic mental image to represent future anxiety-provoking situations. We assume that this can be facilitated if the therapy is centered on specific actions of the patient in the VE, leading us to propose the ACET approach described below.

## 3. Action-Centered Exposure Therapy (ACET) 

Many theories of PTSD agree that PTSD is the result of pathological associations of stimuli with danger [55]. Therefore, it follows that the treatment would involve unlearning these associations and learning new ones [31,53]. We hence refer to learning theories (behaviorist, cognitivist, and constructivist) and recent advances in SGs to design a new approach named ACET: action-centered exposure therapy. ACET is an exposure therapy focused on the patient’s action and interaction within a VE. Unlike cue exposure therapy, we present ACET as a gradual therapy, not in the sense of stimulus intensity, but in the sense of the evolution of the patient’s confidence regarding his environment to facilitate treatment and to maximize effectiveness. Although the emotional processing principle specifies that the fear memory should be activated before anything else [53], as in [51], we argue that this activation should not be too stressful for the patient in order to avoid re-traumatization. Hence, the primary goal of an ACET-based exposure therapy is not only to provoke emotional reactions, emotional reactions [56]. Instead, it aims to replace these emotional reactions with motor ones to help the patient overcome his anxiety without avoidance. This finds its place in the application of behaviorist, cognitivist and constructivist learning theories. Nevertheless, the confrontation with these stimuli is meant to be somewhat indirect, allowing the patient to gain confidence during the exposure [22].

In the ACET context, we link the action of the patient to the active learning methods based on a process of discovery, reflection, and decision making. The patient acquires new skills and new behaviors through his logical construction. This construction involves sensory, physical and cognitive activities, and is extended to the analysis of consequences on the exposure environment. The actions performed are related to the environmental content and to the expectations of the patient. These expectations are “an interpretation of the current situation based on an entire history of previous interactions” [46]. Thus defined, the actions of the patient could have effects at several levels of therapy.

- **Motivation and engagement.** By participating in the task of acquiring new learning during treatment, the patient could be more engaged and could feel more motivated to continue treatment [13] (p. 18). This is an important feature considering the high discontinuation rate in traditional exposure therapies.
- **Observation and perceived self-efficacy.** The action has the great advantage of being observable by both the therapist [22] and the patient. The action allows to the therapist to better understand the state and the progress of his patient [22]. For the patient, the action promotes the perception of his self-efficacy [57] (pp. 1–4).
- **Personalization of the treatment.** Given that action is specific to each individual because it is linked to the notion of reflection and decision making [45], each patient will have the ability to gradually adapt his action to his own therapeutic needs.
- **Presence.** Particularly for the in-virtuo exposure therapy, the feeling of presence from the patient is essential to initiate the treatment processes [13] (p. 27). The action allows the subject to be more present in the virtual environment [58].
- **Strengthening of associations.** Since the aim of the therapy is to facilitate new learning by creating associations between stimuli and the “absence of the problem,” these associations can be facilitated by the action [59]. Learning can be consolidated by the recurrent practice in varying VE contexts being generalized in the patient’s future life (after therapy) [13] (p. 110).

The ACET approach consists of three main stages:

### 3.1. A Trivialization of the Stimuli Associated to the Trauma

It is well known that people suffering from PTSD often excessively use alcohol and/or drugs ([50] (p. 505), [60]). We see this as their effort to cope with the effect of PTSD symptoms. Therefore, we assume that these people are willing to find a means of escape from their everyday symptoms. That is why the first step of our approach is to trivialize the VE that will be used for therapy. At this stage, our goal is to denature the environment to allow the user to be able to appropriate it. Indeed, although a user has been traumatized by a given situation, he can always imagine a trivialized world, a magical environment related to the considered situation in which he will be able to have fun. For example, consider the case of patients suffering from trauma related to a truck accident [22]. Such people can easily have a strong emotional reaction at the mere sight of a truck (even a virtual one). In this case, the magical environment can be a realistic highway where the patient drives a flying carpet. This change of the virtual world has allowed patients to experience a unique gaming experience in which they were pleased to appropriate the game mechanics (the driving) [37]. It is important to note that this stage differs from the first phase that is usually exploited in habituation approaches. Indeed, here the main advantage is that when experimenting in the trivialized environment, the patient is already experimenting with the actions on which his therapy is centered. In the proposed example, the actions are the driving process.

### 3.2. From Fantasy to Reality

Once the patient has enjoyed experimenting with the fantasy environment, this phase aims to bring him into a more realistic universe while exploiting the previously learned actions. In order to do so, the elements of decor proper to the trivialization of the environment must be replaced by their real representations. Thus, in the case example cited above, a real truck replaces the flying carpet. We recognize that the environment at this stage is a potentially anxiety-provoking environment. However, unlike stimuli-focused therapies, our theoretical foundation states that since the patient had an experience like the current situation in the previous step, it is expected that he will cling to the action as his focus. As a result, he is expected to be able to make other non-problematic associations and meanings with the potentially anxiety-provoking elements. At this stage, he should already be starting to distance himself from the post-trauma stimuli.

### 3.3. Indirect Exposure to the Traumatic Situation

The last phase, which is considered the most delicate, involves the indirect exposure of the patient to anxiety-provoking stimuli. Careful monitoring of the anxiety level of the patient is important to prevent re-traumatizing the patient. The idea is to help the patient to understand that he should not deny that the stimuli associated with his trauma are part of life and thus should not be ignored. However, such stimuli are not as problematic as he previously thought. This impact of this confrontation will be perceptible through observable behaviors of the patient. In the case study with the truck-driving simulator, the observable behaviors of the patient at this stage included verbalization and the attempt to avoid the place of his accident (traumatic situation). 

To determine the actions and to better achieve the therapy goals through these three phases, the application of the learning mechanics–game mechanics (LMs-GMs) model proposed in [61] may be beneficial. This paper suggests that the acquisition of knowledge and skills can be achieved through game mechanics (GMs). GMs allow interactions and actions in the game world, the VE [62]. The LMs-GMs model is based on various theories of learning mechanics (LMs) and different interaction methods from the game theory literature. According to this model, the gamification process can effectively concretize the transition of the main LMs to the GMs involved in the process [63]. Such a transition occurs by means of self-directed activity of the subject [64] in the GMs, which can be either new learning included in the mechanics of the game or the use of some reflexes from the subject’s cognitive background (actions to which the player is accustomed) [63,64]. In the ACET-approach context, we propose considering the patient’s profile, the context and the goals of therapy when choosing GMs. In addition, the patient must have significant returns on his actions from the environment [64]. If it is difficult or impossible to create GMs by exploiting the patient’s cognitive background, we propose GMs to be either directly included in the new learning that one wants to transmit to the patient or be easy to learn so as not to complicate the main learning of the therapy. For example, exploiting haptic feedback could facilitate the patient’s learning in the virtual environment [65].

## 4. Empirical Case 

The proposed approach has been implemented in our previous work and detailed in [22]. This study focuses on a former truck driver who was suffering from PTSD following an accident. After a tire puncture, his truck turned over and caught fire, at which point he lost consciousness and then awoke to hearing a driver shouting to him to shelter himself for fear of explosion. Since this accident, the patient, who had been a truck driver for 20 years, became inactive. He was continually anxious and depressed. He was followed by a psychiatrist and was under medication. 

The ACET-based treatment was integrated into a counseling center. The proposed simulator was developed to be customizable for many patient’s profiles. The VE provides both stimuli related to stressful situations for a truck driver and various possibilities of action. These two components were studied through two processes: gamification and customization of the truck driving simulator.

### 4.1. Procedures

The gamification process was carried out by implementing links between the game mechanics and learning mechanics from the LM-GM model presented in [61]. LMs-GMs associations that were considered and their implementation are listed in the Table 1.

The customization process was programmed to give the possibility to create, for many patient profiles, a VE almost identical to the traumatic environment able to evoke emotions and to offer flexible scenarios [21]. Thus, an environment editor that is integrated directly into the “Unreal Engine 4” game engine has been developed to be easy to use for the supervisor. To do so, the focus has primarily been on managing the personalization of roads because the road is the key element for a trucker in the execution of his task. Second, anxiety-provoking stimuli were designed based on the traumatic scenario. This was done by first considering the accident scenarios involving vehicles other than the truck driver’s vehicle and whose severity can be adapted to the mental state of the patient, followed by the loss of control commonly due to adverse weather. A tool to monitor the time and the weather has been provided for this purpose. Additionally, the virtual truck could be customized by the patient to resemble his actual truck. To optimize the customization process, a generic artificial intelligence was developed for the management of elements other than those handled by the patient or by the supervisor. In addition, there was an option in the game to attach trailers to the patient’s truck.

### 4.2. Materials

Various devices have been exploited to create an immersive but low-cost truck-driving simulator. The immersive aspect has primarily been managed by a head-mounted display (Sony HMZ-T2/T3, Sony, Tokyo, Japan) and a device containing both the steering wheel, gearbox and pedals (Logitech G27 3 Driving Force GT Racing Wheel, Logitech, Apples, Swiss). For the Sony HMZ-T2/T3, an interface has been developed with the NaturalPointTrackIR 5 headtracking system (NaturalPoint, Inc., Corvallis, OR, USA) to reflect a sense of realism. As in reality, a wheel (the Logitech G27 3 Driving Force GT racing wheel, Logitech, Apples, Swiss) was used to move the truck and offered other basic features such as lights, sounds, and wipers.

### 4.3. ACET-Based Treatment

The ACET-based treatment was gradually extended over eight sessions grouped in three stages as follows.

#### 4.3.1. Trivialization of the Stimuli Associated with the Truck Driver’s Trauma

The first two sessions were devoted to pre-exposure of the truck driver using an allegory: the flying carpet. The carpet was controlled using the helmet and steering wheel, and the actions remained the same as those of driving. This stage allowed the patient to become familiar with his exposure environment and to reconnect with driving mechanisms without direct exposure to a truck.

#### 4.3.2. From Fantasy to Reality 

This stage took place in Sessions 3, 4, and 5 of the treatment. The third session was the first exposure to the actual truck. The patient had to walk around a virtual parking lot with several trucks and choose the one that he would eventually drive. He intervened with himself during his therapy, and his choice of vehicle was the main action of this session because it symbolized a first step toward acceptance of driving his chosen truck and testified to the engagement of his actions in his therapy. In that session, the indirect anxiety-provoking stimuli were trucks, especially trucks resembling the one involved in the patient’s trauma. Sessions 4 and 5 consisted of driving this truck freely, without being exposed to an accident, but in varying weather conditions. Here again, the patient was constantly active: he spoke, switched on the radio, resumed habits of observable behaviors, and the therapist observed his evolution and his avoidance strategies. His actions represented his engagement, his motivation, and his presence in the VE as he reconnected with his rational reflexes and driving skills and with the pleasure of driving. The indirect stimulus here was the conduct and the routes that the patient knew, including the one on which his accident took place and which he tried to avoid. The patient controlled the environment and acted to advance his treatment; he did not give up and did not lose control of the situation or his lucidity toward it. The treatment evolved by adding a towing mission to the driving. Once again, the session was characterized by a certain number of actions and witnessed reactions that demonstrated his concentration and feelings toward his accomplishments and his stress. Changes in his driving after towing still illustrated his immersion and his “behavioral presence” in the scenario, as he felt he had a heavier load and therefore drove more slowly and with more precautions. 

#### 4.3.3. Indirect Exposure to the Traumatic Situation (A Burning Truck)

The sixth session indirectly exposed the patient to the trauma and consisted of passing a burning truck on the roadside. The patient reacted by exteriorizing and verbalizing excessively and ostentatiously. He refused to stop at the scene of the accident and agreed to continue driving his truck. This way of acting and interacting with his trauma showed that despite his avoidance behaviors, the patient managed to retain control and to choose a rational outcome. This was a positive progress in the treatment. For the last two sessions, as at the fourth session, the emphasis was again placed on the driving mechanisms. 

At the end of the treatment, the driver reconnected to reality and was then able to realize that he no longer intended to drive a truck and could undertake professional retraining. This awareness is synonymous with successful treatment because the former driver was able to become active and resume the course of his normal life. The therapy allowed him to step back from his trauma and reconnect with his identity and the reality of his condition, dissociating himself from his truck with which he had learned to be embodied. He also agreed not to be heroic and invincible. These shifts meant he could then “move on to something else” and “take control.” 

Each stage of the game allowed the patient to reconnect with his abilities and emotions and allowed the therapist to observe the externalization of patient’s improvements and emotions and then draw conclusions to exploit them. For example, when choosing a truck at the beginning of the treatment, the patient had never chosen a truck like the one he was driving, but at the end of the treatment, he finally selected a red truck like his own. This choice, which reflected an improvement in the patient’s emotional state regarding his trauma, his beliefs, and dysfunctional behaviors, was directly observable by the therapist. The patient did not display any reluctance to take the virtual truck to the next stage. The action of the subject and indirect stimuli related to his trauma was the focal point of each stage. In this case study, all aspects of ACET are clear: indirect and progressive exposure to anxiety-provoking stimuli, subject’s free will, presence and immersion of the subject, engagement and motivation, observable and representative externalization of emotions and behaviors, re-appropriation of behavioral skills, perception of progress and achievements, behavioral and cognitive rehabilitation, and finally reparation and re-education of the link with reality and with one’s own identity.

## 5. Conclusions

According to learning theories, a passive systemic exposure to traumatic stimuli should not be the best option for the treatment of PTSD. In this paper, we described the theoretical foundations of a new approach: ACET. It focuses on the action and activity of the patient in his or her exposure environment. ACET also promotes active learning of safer and healthier coping strategies combined with systematic exposure to reduce the psychological distress associated with PTSD. For this, ACET exploits advanced interactions and gamification techniques to allow the user to be active in an environment centered on learning. This approach contains three main phases. The first is dedicated to the trivialization of the stimuli associated to the trauma, the goal being to denature the environment to allow the user to be able to appropriate it. Doing so, this stage allows the user to experiment with the actions on which the therapy is centered. The second stage brings the patient into a realistic universe. In this potentially anxiety-provoking environment, it is expected that the patient will cling to the action as his center of interest and thus eventually be able to make other non-problematic associations with these stimuli. In the last stage, there is an indirect exposure to the anxiety-provoking stimuli. We believe that with the ACET approach, the therapist will have a better idea of the knowledge, meaning, and explanations that the patient gives to his anxiety and be better able to guide him during treatment. This paper has also described the first step for the validation of this new approach; more experiments are needed. In the near future, we plan to run more studies. Doing so, we will eventually find some limitations. Moreover, it would be interesting to evaluate the usefulness of an ACET-based treatment for other mental disorders.

## Figures and Tables

**Table 1:** Game and learning mechanics of the simulator, based on the LM-GM model.

	Game Mechanics	Learning Mechanics	Implementation
1	Movement, Simulate/Response, and Realism	Explore, Simulation, and Action/Task	Immersion through an HMD and a racing wheel
2	Levels, Cascading Information, and Behavioral Momentum	Repetition	Levels
3	Story and Information	Guidance and Instructional	Radio Messages and Level Conditions (job given)
4	Tokens		World Events: Weather, Day Time, Car Crashes
5	Feedback, Rewards, and Status	Feedback and Motivation	Level Change, Pop-up, and Truck Customization
6	Design/Editing	Ownership	Customization of the Truck
